# Efficacy and Safety of Cannabidiol Cannabis Extracts for Children and Adolescents with Autism Spectrum Disorder: A Systematic Review and Meta-Analysis of Randomized Controlled Trials

**DOI:** 10.1192/j.eurpsy.2025.369

**Published:** 2025-08-26

**Authors:** L. Cappelleti Beneti Branco, I. Borja De Oliveira, G. A. M. Alves, M. Teranishi, D. Xavier, E. Sávio Schneider Motta, F. Wagner, M. L. Geremias, A. V. de Vasconcelos, A. C. Gonçalves Tames Zambrana, J. P. De Aquino

**Affiliations:** 1 São Camilo University Center; 2 School of Medicine, University of São Paulo, São Paulo, Brazil; 3 Humanitas University; 4 Università degli Studi di Milano, Milan, Italy; 5 ECPE - PPCR Program, Harvard T. H. Chan School of Public Health, Boston, United States; 6 Federal University of Espírito Santo, Espírito Santo; 7 School of Medicine, Pontifical Catholic University of Rio Grande do Sul, Porto Alegre; 8 University of the Joinville Region, Joinville; 9 Afya College of Medical Sciences of Santa Inês, Santa Inês; 10 Zambrana Clinic, Itajubá, Brazil; 11 Department of Psychiatry, Yale University School of Medicine, New Haven; 12 VA Connecticut Healthcare System, West Haven; 13 Clinical Neuroscience Research Unit, Connecticut Mental Health Center, New Haven, United States

## Abstract

**Introduction:**

Autism Spectrum Disorder (ASD) affects approximately 3% of children and adolescents in the U.S. This condition is increasingly prevalent worldwide and presents significant treatment challenges. Preliminary evidence suggests that cannabidiol (CBD) cannabis extracts may help manage ASD symptoms, but their efficacy and potential harms have not yet been systematically investigated.

**Objectives:**

To systematically review and meta-analyze the evidence from clinical trials investigating the efficacy and safety of CBD cannabis extracts in alleviating symptoms of ASD in children and adolescents.

**Methods:**

We conducted a comprehensive search in MEDLINE, Embase, PsycINFO, and the Cochrane Central Register of Controlled Trials using MeSH terms including “Autism Spectrum Disorder,” “Cannabidiol,” “Cannabis,” “Child,” and “Adolescents.” No language or publication date restrictions were applied. The search was last updated on September 8, 2024. We included randomized, placebo-controlled trials on the efficacy or safety of CBD cannabis extracts in children and adolescents with ASD. For outcomes with limited study data, we used a fixed-effects model. The risk of bias in the included studies was evaluated using the Risk of Bias 2 tool.

**Results:**

Three studies met our criteria, comprising 276 participants (78.3% male; mean age 10.5 years, range 5 to 21). Interventions included orally administered CBD cannabis extracts, with tetrahydrocannabinol (THC) present in minimal amounts or in ratios of 9:1 to 20:1 CBD to THC. Dosages of CBD started at 1 mg/kg per day and were titrated up to 10 mg/kg per day. CBD cannabis extracts significantly enhanced social responsiveness (SMD = -0.75 [-1.08, -0.43], p < 0.01, I² = 17%), reduced disruptive behavior (SMD = -0.36 [-0.67, -0.06], p = 0.02, I² = 0%), and alleviated anxiety (SMD = -0.33 [-0.63, -0.03], p = 0.03, I² = 59%). CBD cannabis extracts also improved sleep quality, without reaching statistical significance (SMD = -0.19 [-0.49, 0.11], p = 0.21, I² = 0%). There was no significant difference in adverse effects between interventions and placebo (odds ratio = 2.11 [1.00, 4.46], p = 0.05, I² = 38%).

**Conclusions:**

CBD cannabis extracts appear to provide meaningful benefits for children and adolescents with ASD, showing moderate improvements in social responsiveness and small yet notable reductions in disruptive behaviors and anxiety. They do not seem to significantly increase adverse effects compared to placebo, suggesting a favorable safety profile. These findings support the potential consideration of CBD cannabis extracts in ASD treatment plans. However, the review’s limitations include a small number of studies, limited sample sizes, and significant heterogeneity. Future research with larger, robust trials is needed to clarify the efficacy and safety of CBD cannabis extracts in managing ASD.

**Disclosure of Interest:**

None Declared

